# An Object Lesson in Public Water Services

**Published:** 1898-12-24

**Authors:** 


### An Object Lesson in Public Water Services.

Among the many very interesting subjects dealt with in the recently issued report of the Medical Officer to the Local Goverment Board few are of such importance from the sanitary point of view as the account of an inspection of the water supply of Bury made by Dr. Bruce Low. Bury is an important manufacturing town, the waterworks of which are owned by the corporation, and supply not only the town itself but various outlying districts, the total population drawing from them numbering 157,500. For a series of years repeated complaints have been made to the Board in regard to the quality of the water supplied by the corporation, and, with the report before us as to the conditions existing on the gathering grounds, we are not surprised. The wonder is that the district has not been ravaged by epidemics. The supply is derived from six gathering grounds, varying from 202 to 3,313 acres in extent, and there are nine impounding or service reservoirs. On one of the gathering grounds, covering 872 acres, there were houses containing 128 persons, the means of excrement disposal being at times privies discharging down the adjacent slopes. Besides this, slop water, tricklings from manure heaps, and the washings after rainfall from lands manured at times with privy contents were ascertained to flow in the direction of and into a reservoir, either directly or by means of rivulets discharging into it. On another gathering ground Dr. Low counted 61 privies and 35 tub-closets, some of the privies discharging on to dung-heaps, the fluid filth from which soaks into the grass or trickles downhill towards the watercourses. Moreover, although tub-closets seem to have been largely introduced into the district in place of the old middens, the most abominable recklessness prevails in regard to the disposal of the matters so collected. Indeed, so far as the disposal of excrement is concerned, the district would seem to be in a state bordering on savagery. "For instance," says Dr. Low, "near a couple of cottages I saw five heaps of tub-closet filth 'dumped' down on a grassy slope within a few yards of a rill on its way to the reservoir, which was only 400 yards distant. At another place I saw a steep meadow slope which had recently been top-dressed with human excrement at a point only 18 yards from the main tributary to the reservoir. A privy midden, open, wet, and foul, was seen discharging on the sloping surface only 11 yards from the flowing water, and a large pool of black slop water was seen soaking to the stream, only 8 yards distant. . . . At the time of my visit I saw, situated only 130 yards from the main brook flowing to the reservoir, a meadow, a portion of which, measuring 114 yards by 40 yards, had been recently heavily top-dressed with tub closet filth. It needed only heavy rain to wash a large part of this mass of human excrement down the surface of the slope to the stream and thence into the reservoir." The high road from Haslingden to Blackburn passes through the whole length of this gathering ground, running at a distance of from 150 to 300 yards away from the reservoir. "Frequent evidence was met with showing that the roadsides, or the fields adjoining the road, were commonly used by workmen and others as places for defæcation." Further on it is said that "a field close to the works was thickly littered in places with human stools, the field being apparently preferred to the privies by the workmen." But we need not continue these nauseating details beyond mentioning that on the same gathering ground there are two burial grounds, one within 400 yards of the stream, which is the main feeder to the reservoir, that in it the ground is so wet that when graves are dug water often has to be baled out, and that on the sloping meadows between the graveyard and the reservoir at least two springs come to the surface. No doubt the other gathering grounds are not so bad as the one picked out for description. But that does not matter. Even if the other sources of supply were absolutely pure, that would not lessen the abominations which are described in the report before us, and unfortunately the other sources, although not presenting quite such flagrant instances of pollution, are by no means beyond reproach, being, in fact, tainted in the same way although in less degree.

Now comes the point. All this water is distributed unfiltered to the inhabitants of the district! "On leaving some of the collecting reservoirs the water passes through a copper wire gauze screen before it enters the mains; the larger solid particles contained in the water are thus prevented from passing into the pipes. But the screening, when practised at all, is insufficient. Occasionally, from exceptional pressure of the outflowing water the screen or strainer is ruptured, with the result that minnows, newts, and the like gain entrance to the mains and create alarm or disgust in the minds of the householders when such unwelcome visitors are delivered from their taps. Occasionally smaller creatures like the water flea can be easily recognised by the naked eye in the water drawn from the house taps." Now we do not wish to gird unduly at municipalisation, for indeed we feel strongly that if all electors took an intelligent interest in town affairs such a mode of ownership, as applied to various undertakings, might be quite ideal. But in daily life we have to take men as they now stand, and we cannot shut our eyes to this fearful example, which should prove that municipal ownership of a water supply gives no guarantee of either purity or good management.

